# Preventing regrettable substitution through regulatory frameworks: using REACH regulation and Stockholm Convention as case studies

**DOI:** 10.1186/s12302-026-01470-0

**Published:** 2026-07-22

**Authors:** Olasunkanmi Dosunmu, Rob Whiting, Avtar S. Matharu, Nigel Watson, Andrew J. Sweetman

**Affiliations:** 1 Lancaster Environment Centre, Lancaster University, Lancaster, LA1 4YQ UK; 2 WSP, Reading, RG1 3EU UK; 3 Green Chemistry Centre of Excellence, Department of Chemistry, University of York, York, YO10 5DD UK

**Keywords:** REACH, Stockholm Convention, Chemical sustainability, Substances of very high concern (SVHC), Persistent organic pollutants (POPs), Regrettable substitution, Hazardous substances, Reflexive thematic analysis

## Abstract

**Background:**

Regrettable substitution, which involves the replacement of a hazardous chemical with an alternative that later proves equally harmful or introduces new risks, remains an ongoing significant challenge for chemical regulation. As regulatory frameworks such as REACH (Registration, Evaluation, Authorisation and Restriction of Chemicals) and the Stockholm Convention increasingly drive transitions away from the use of hazardous substances, the need to prevent regrettable substitution has become central to effective chemicals management.

**Methods:**

This study examines how regrettable substitution can be prevented through deliberate regulatory design and implementation. Using REACH and the Stockholm Convention as case studies, we performed a qualitative study involving interviews and document analysis.

**Results:**

Reflexive thematic analysis generated interconnected themes that collectively identify how regulatory frameworks can potentially prevent regrettable substitution. These include improving the reliability, completeness, and transparency of regulatory data to support informed substitution; using incentives and structured regulatory support to promote innovation in safe(r) alternatives; establishing coherent and coordinated regulatory approaches that reduce fragmentation; strengthening partnerships among regulators, industry, and scientific actors to enhance information flow; adopting proactive and precautionary regulatory strategies, including early screening; and reinforcing compliance through enhanced monitoring and enforcement.

**Conclusions:**

The findings demonstrate that regrettable substitution is not merely a scientific or technical failure but a systemic and operational regulatory issue arising from late hazard identification, incomplete exposure and performance information, siloed regulatory structures, and uneven innovation incentives. The study concludes that preventing regrettable substitution requires regulatory frameworks that are anticipatory, integrated, transparent, and adaptive that can actively steer the substitution process toward genuinely safe(r) and sustainable chemical solutions.

## Background

The expansion of chemical production following the industrial revolution in Europe (c. 1750–1850) marked the beginning of a growing societal reliance on chemicals [64, 75, 79]. Today, chemicals are deeply embedded in modern life, underpinning technological development, economic growth, and societal wellbeing. They also play a central role in achieving several United Nations Sustainable Development Goals (UN SDGs), including zero hunger (SDG 2), good health and wellbeing (SDG 3), clean water and sanitation (SDG 6), responsible consumption and production (SDG 12), and climate action (SDG 13) [95]. Yet the same properties that make many chemicals useful also create significant concerns. Uncontrolled production, widespread use, and the introduction of novel chemistries have been associated with human health concerns, biodiversity loss, persistent environmental contamination, and potential disruption of planetary systems [40, 56, 60, 68, 77].

These concerns are particularly acute for chemicals identified as Substances of Very High Concern (SVHC) under REACH (Registration, Evaluation, Authorisation and Restriction of Chemicals) Article 57 such as carcinogens, mutagens and reprotoxic (CMR Cat 1A/1B), persistent, bioaccumulative and toxic (PBT), (very) persistent and (very) bioaccumulative ((v)P(v)B, and those of equivalent concern (EqC) (e.g., endocrine disruption) and for persistent organic pollutants (POPs) under the Stockholm Convention, which are characterised by Long‑Range Environmental Transport (LRET) [21, 86]. Both REACH and the Stockholm Convention were implemented to address these serious risks and protect human health and the environment [91].

**Fig. 1:**
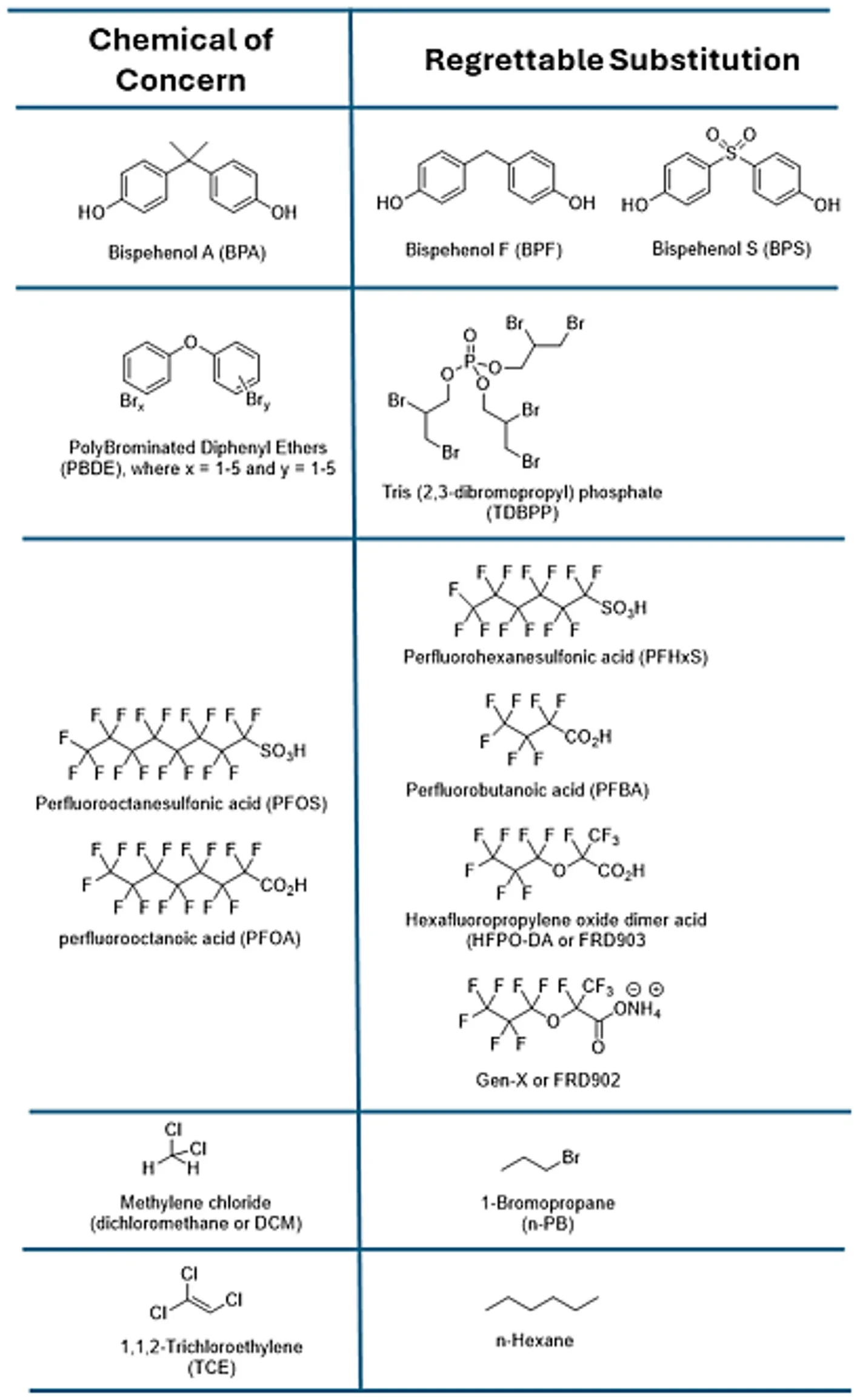
Examples of regrettable substitutions

Chemical regulations such as REACH and the Stockholm Convention therefore perform a crucial role in controlling hazardous substances and in driving their substitution with safe(r) alternatives [3, 19, 27, 59, 69, 72, 74, 80, 83, 91, 101]. The Stockholm Convention lists chemicals for global restriction or elimination based on their hazard profiles of persistence, bioaccumulation, toxicity and LRET [87], while REACH regulates SVHCs and EqC substances through authorisation, restriction, or bans [21, 62]. While there is partial overlap particularly for POPs that are also regulated under REACH, the scope and coverage of the two frameworks differ. The Stockholm Convention targets a relatively limited set of globally prioritised substances with specific hazard profiles, whereas REACH has a broader scope, applying to a much wider range of chemicals placed on the EU/UK market and addressing multiple risk management pathways across diverse uses. Allowing for many hazardous chemicals to be replaced with alternatives; however, instances of regrettable substitution, the hazard replacement of one harmful chemical with another that is equally harmful or where the impact of the chemical is shifted from one concern to another are well documented. Examples include (see Fig. 1) the substitution of bisphenol A (BPA) with bisphenol F (BPF) and bisphenol S (BPS); polybrominated diphenyl ethers (PBDEs) with organophosphate flame retardants (OPFRs) such as tris(2,3-dibromopropyl) phosphate (TDBPP); perfluorooctane sulfonic acid (PFOS) and perfluorooctanoic acid (PFOA) with alternatives like perfluorohexane sulfonic acid (PFHxS), perfluorobutanoic acid (PFBA), and hexafluoropropylene oxide dimer acid (HFPO-DA or FRD903, with its ammonium salt known as GenX or FRD 902); methylene chloride (dichloromethane or DCM) with 1‑bromopropane (n-PB); and 1,1,2-trichloroethylene (TCE) with n‑hexane.

These substitutes initially introduced as safer alternatives, have themselves raised concerns, perpetuating cycles of harm or negative impact rather than resolving them [5, 9, 17, 19, 66, 76, 82, 84, 104]. Since chemical regulations are major drivers of chemical substitution, shaping its timing, direction, and nature; under certain conditions, frameworks such as REACH and the Stockholm Convention may unintentionally contribute to regrettable substitution. This recognition is essential for improving the effectiveness of regulatory frameworks. For instance, Dosunmu et al., [20] critically examine whether and how EU/UK REACH and the Stockholm Convention may unintentionally exacerbate regrettable substitution. Their findings suggest that regrettable substitution is not merely an unintended outcome of isolated decision-making but is structurally embedded within regulatory systems. Specifically, regrettable substitution emerges from the interaction of systemic and operational constraints, including limited and uneven data availability on alternative substances, fragmentation across regulatory frameworks and jurisdictions, and time pressures that prioritise regulatory compliance over meaningful innovation. In addition, barriers to information flow can restrict the effective evaluation and comparison of alternatives. Taken together, these factors create conditions in which substitution decisions are made under uncertainty, thereby increasing the likelihood that alternatives may replicate or introduce similar risks rather than delivering genuinely safe(r) outcomes. This article therefore examines how regrettable substitution can be prevented through more robust, coherent, and proactive regulatory frameworks, using REACH and the Stockholm Convention as case studies. Preventing regrettable substitution is critical not only because chemicals play a fundamental role in achieving the UN SDGs and in delivering a greener, more sustainable Europe, including the ambitions of the European Union (EU) Green Deal to achieve climate neutrality by 2050 [27, 43]. Also, because the repeated cycle in which hazardous chemicals are replaced by equally harmful alternatives must not continue. By analysing the working of REACH and the Stockholm Convention, this study provides insights into how regulatory design and implementation can reduce the likelihood of regrettable substitution and support the transition toward truly safe(r) and sustainable chemical use.

## Methodology

This study employed a qualitative research design combining document analysis with semi‑structured interviews with key stakeholders to explore how REACH and the Stockholm Convention can be better implemented to prevent the occurrence of regrettable substitution. Participants were asked the question among others: “Given your experience, how might you suggest chemical regulations could be improved or strengthened to reduce the possibilities of regrettable substitutions? Can you give an example of how that might work?” This question was designed to elicit practical, experience-based insights into regulatory gaps, potential improvements, and real-world examples of how regulatory frameworks such as REACH and the Stockholm Convention could more effectively prevent regrettable substitution. A total of 31 semi-structured interviews were conducted via Microsoft Teams between November 2024 and March 2025. The number of interviews was shaped by participant availability and the time constraints for the research, while ensuring inclusion of a diverse range of perspectives across relevant sectors. In parallel, policy, regulatory, and guidance documents were analysed, with primary focus on REACH and the Stockholm Convention, see Table 1 for the list of documents analysed. Documents were selected purposively based on their relevance to key elements of REACH and the Stockholm Convention, and their relevance to chemical substitution. The sample includes regulatory texts, policy documents, and technical guidance covering core aspects such as registration, authorisation, and restriction, as well as EU policy such as the Chemicals Strategy for Sustainability (CSS), the European Green Deal, and SSbD addressing the reduction of pollution from hazardous chemicals.

**Table 1:** List of documents analysed

Regulatory documents
Regulation (EC) No 1907/2006 of the European Parliament and of the Council of 18 December 2006 concerning the Registration, Evaluation, Authorisation and Restriction of Chemicals (REACH), establishing a European Chemicals Agency, amending Directive 1999/45/EC and repealing Council Regulation (EEC) No 793/93 and Commission Regulation (EC) No 1488/94 as well as Council Directive 76/769/EEC and Commission Directives 91/155/EEC, 93/67/EEC, 93/105/EC and 2000/21/EC (REACH Regulation EC No. 1907/2006) [41]
Regulation (EC) No 1272/2008 of the European Parliament and of the Council of 16 December 2008 on classification, labelling and packaging of substances and mixtures, amending and repealing Directives 67/548/EEC and 1999/45/EC, and amending Regulation (EC) No 1907/2006 (Classification, Labelling and Packaging (CLP) Regulation) dated Dec. 2023 [47]
Proposal for a Regulation of the European Parliament and of the Council establishing a common data platform on chemicals, laying down rules to ensure that the data contained in it are findable, accessible, interoperable and reusable and establishing a monitoring and outlook framework for chemicals [48]
Commission Implementing Regulation (EU) 2020/1435 of 9 October 2020 on the duties placed on registrants to update their registrations under Regulation (EC) No 1907/2006 of the European Parliament and of the Council concerning the Registration, Evaluation, Authorisation and Restriction of Chemicals (REACH) [44]
Treaties, Agreements and related Documents UNEP: Stockholm Convention on Persistent Organic Pollutants [89]
Stockholm Convention on Persistent Organic Pollutants (POPs) Texts and Annexes: Revised in 2023 [92]
**Policy documents**
WHITE PAPER Strategy for a future Chemicals Policy [40]
The European Green Deal [43]
Chemicals Strategy for Sustainability (CSS) Towards a Toxic-Free Environment [45]
Safe and sustainable by design chemicals and materials: Revised 2025 [58]
**Guidance documents**
Guidance on the preparation of an application for authorisation [30]
Guidance on the preparation of socio-economic analysis as part of an application for authorisation [26]
Guidance for the preparation of an Annex XV dossier for restrictions [25]
Generic Exemptions from the Authorisation Requirement [28]
How to apply for authorisation [31]
Changes of market volumes of chemicals subject to authorisation in 2010–2021 [33]
Guidance on registration [32]
Report of the POP Review Committee: General Guidance on considerations related to alternatives and substitutes for listed persistent organic pollutants and candidate chemicals [90]

A purposeful sampling strategy was used to identify individuals with direct professional experience in chemical regulation, risk management, and chemical substitution. Participants were drawn from key stakeholder groups, including government (regulatory and policy), non‑governmental organisations (NGOs), trade associations, consultancies, academia, and industry. To further broaden participation, a snowball sampling technique was used, whereby interviewees recommended additional individuals within their professional networks who met the inclusion criteria. Before each interview, participants received detailed information about the study and provided informed consent. All interviews were recorded, transcribed, anonymised, and cleaned, that is identifiable information removed before analysis to ensure confidentiality and data integrity.

Purposive sampling and voluntary participation introduce limitations, including self-selection and response bias, potentially narrowing the range of perspectives captured. Qualitative interviews are not statistically representative and cannot support probabilistic generalisation but provide in-depth, context-specific insights supported by the inclusion of diverse participants and triangulation. More so, the purposive study design limits the findings to REACH and Stockholm Convention.

Following transcription, both interview data and documents were imported into NVivo (version 15.2.2, https://lumivero.com/) to support systematic organisation and coding. Analysis followed reflexive thematic analysis as developed by Braun and Clarke [10–13]. Reflexive thematic analysis is a theoretically flexible method for identifying, analysing, and interpreting patterns across qualitative data [13]. It is especially suited to studies focused on subjective understanding, meaning-making, and stakeholder perspectives, making it an appropriate approach for this study. Importantly, reflexive thematic analysis positions the researcher as an active, interpretive agent in the development of themes, acknowledging that meaning is co-constructed through iterative engagement and reflexivity with the data [11, 13, 14].

**Fig. 2:**
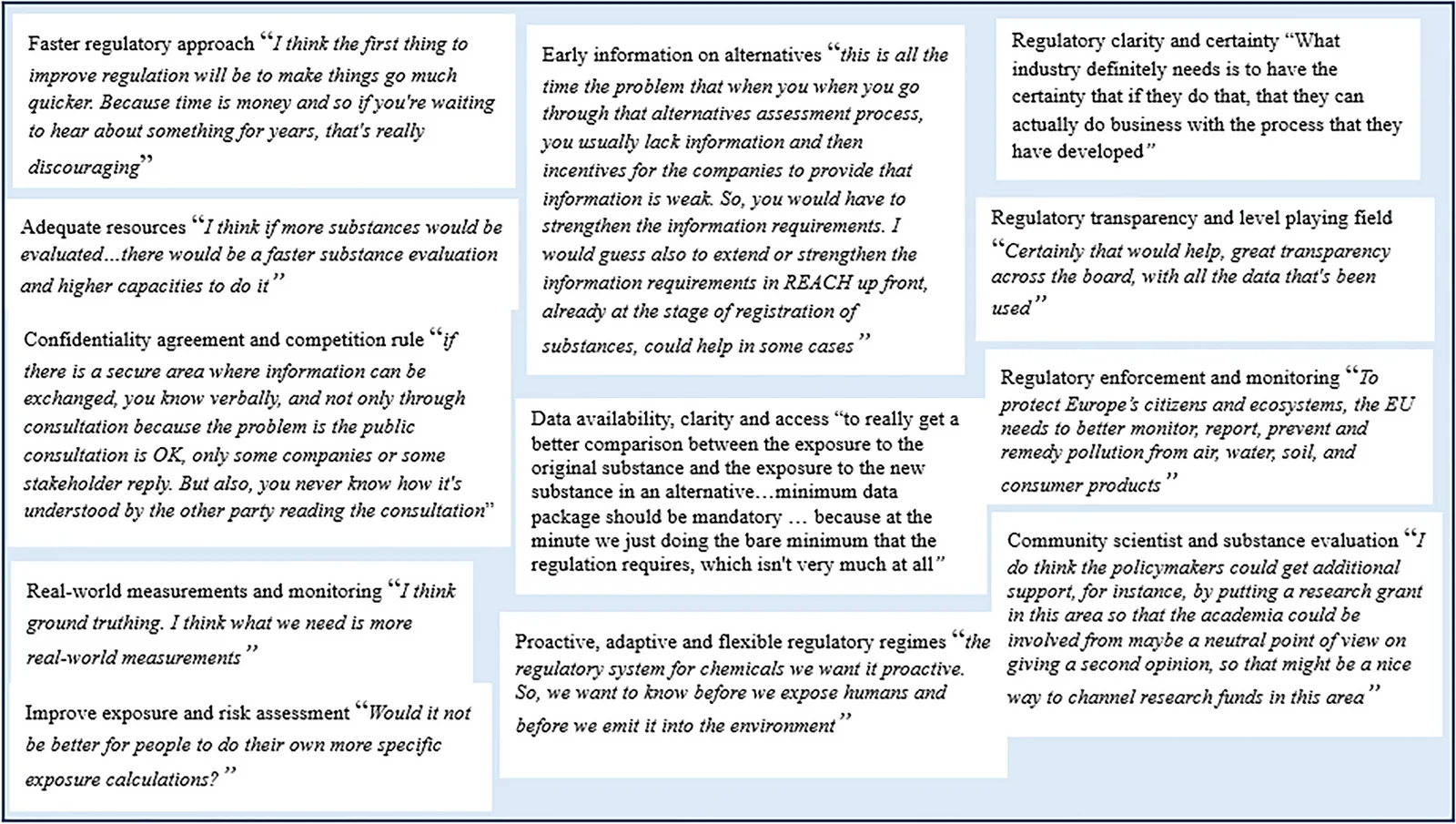
Example codes and quotations

The analysis followed Braun and Clarke’s six‑phase iterative process; in Phase 1 (Familiarisation), interview summaries were prepared, transcripts reviewed and cleaned, and initial engagement with the dataset began, focusing on semantic meanings. In Phase 2 (Systematic Coding), data were coded inductively within NVivo, generating concise labels that captured recurring ideas and patterns. Coding proceeded in two rounds to strengthen interpretive judgement and enhance rigour. NVivo served as an organisational tool; however, the coding process itself remained researcher‑driven and reflexive, see Fig. 2 for example of codes and quotations.

Phase 3 (Generating Initial Candidate Themes) involved exporting codes into Microsoft Excel for clustering into early groups, using semantic and latent coding to explore connections and shared meanings. Colour‑coding was used to sort related codes and identify shared conceptual meanings, ultimately forming initial candidate themes. In Phase 4 (Reviewing Themes), the candidate themes created in Phase 3 were evaluated against both the coded dataset and the full dataset. Themes that lacked coherence or alignment with the research aims were modified or discarded. Phases 5 and 6 (Defining, Naming, and Writing Up Themes) overlapped, refining theme definitions, finalising names, and writing descriptions that clarified central meanings and boundaries. This process produced a thematic map and laid the foundation for reporting the findings. Throughout, transparency and rigour were maintained through systematic engagement with the data, reflexive interpretation, and active theme construction, enabling nuanced insights into the data.

## Results

The findings presented in this section are derived from the systematic analysis of the combined interview and document dataset. Each theme represents the outcome of an iterative reflexive thematic analysis, as outlined above, where codes were progressively developed, grouped, and refined to capture patterned meanings across the data. The resulting themes reflect the researcher’s interpretive synthesis of these patterns, highlighting how regrettable substitution can be prevented. The implications of each theme are explored further in the Discussion section.

### Participant demographics

**Fig. 3:**
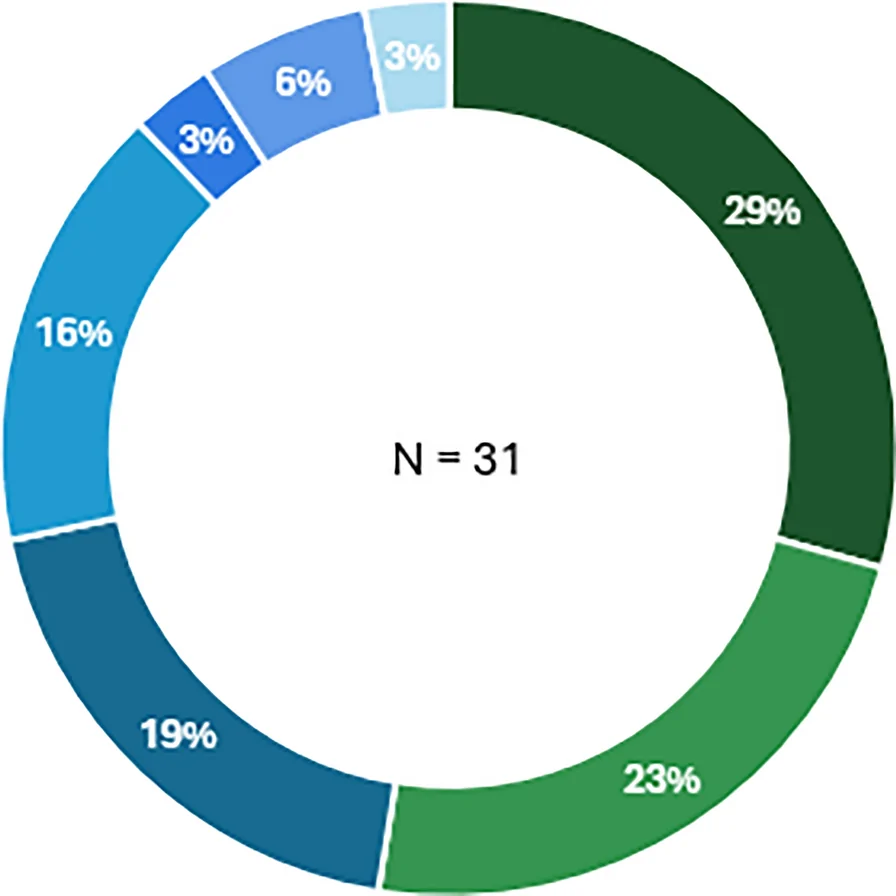
Demographics of semi-structured interview participants

Participant demographics are presented in Table 2 and Fig. 3. The breakdown shows an even representation across the demographics, participants from industry covers chemical, agrochemical, pharmaceutical and consumer good companies.

**Table 2:** Demographic information for interview participants; where, Other = Professional body

Participant sector	Interview (N = 31)	
	Frequency	Percentage (%)*
Industry	9	29
Government (policy and regulators)	7	23
Consultancy	6	19
Academia	5	16
Association	1	3
NGO	2	6
Other	1	3

^*^Total may not exactly equal 100% due to rounding up

### Qualitative analysis findings

**Fig. 4:**
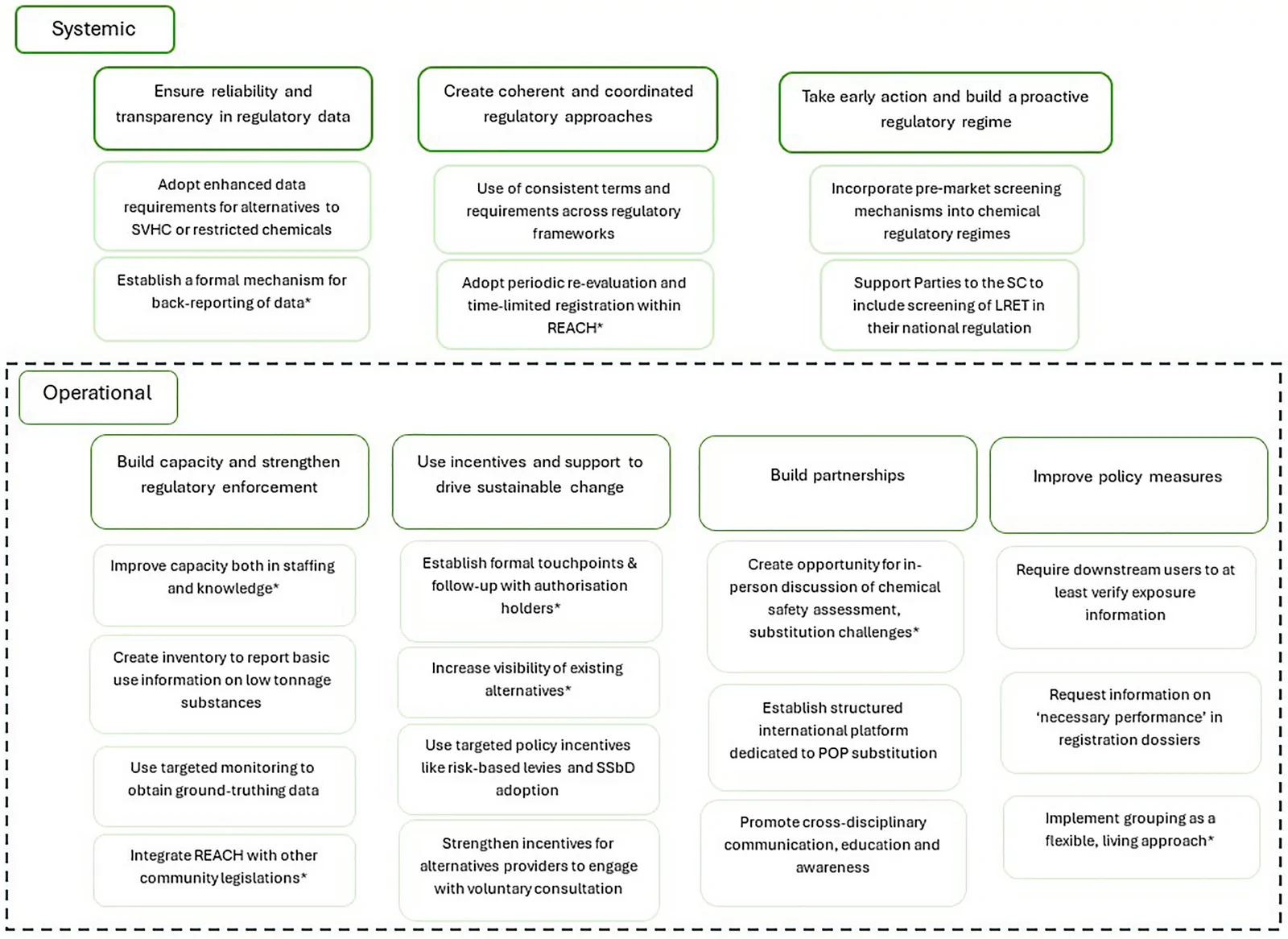
Thematic findings showing how regrettable substitution can be prevented. The dotted line indicates how systemic shape the regulatory context within which operational findings occur. The darker line represents the themes/findings, and the lighter line represents our recommendations corresponding to each theme/finding. *Recommendations that can be adopted quickly based on their regulatory feasibility and alignment with ongoing developments within EU REACH and related policy frameworks

Figure 4 presents the thematic findings illustrating pathways for preventing regrettable substitution. The themes are organised into systemic (challenges embedded within the overall structure, design, and logic of the regulatory system) and operational (challenges relating to the implementation, execution, and day-to-day functioning of regulatory systems) categories, reflecting the structure of, and directly addressing, the findings from our companion study [20]. The figure also incorporates our corresponding recommendations aligned with each thematic area.

## Discussions

Many hazardous substances are substituted as a result of regulatory action, ensuring that chemicals on the market and used by industry and consumers are without negative impacts on human health or the environment. The findings from our thematic analysis (Fig. 4) discussed below reinforce how regulatory frameworks can ensure effective and efficient risk management of hazardous chemicals, promote informed substitution and reduce unintended consequences. The recommendations presented in this study are researcher-driven and were developed through interpretive engagement with the data using reflexive thematic analysis. They do not represent direct or consensus-based proposals from specific stakeholder groups. Instead, they reflect the researcher’s synthesis of patterns and insights identified across both interviews and documentary sources. As such, the recommendations should be understood as analytically derived and data-grounded interpretations, situated within the context of REACH and Stockholm Convention, but not attributable to individual participants or specific stakeholder positions.

### Ensure reliability and transparency in regulatory data

To ensure the development of safe(r) alternatives, high quality, complete, and transparent regulatory data are important elements. When substitution decisions are based primarily on the specific hazard(s) that triggered regulatory concern, such as endocrine disruption, persistence, or carcinogenicity, there is a substantial risk of regrettable substitution. This narrow focus often becomes a proxy for data collection and evaluation rather than a comprehensive hazard assessment [66]. Substituting a well‑characterised chemical with one that has only been tested for the original concern, or with one that has been less extensively assessed, can lead to risks shifting to other endpoints. This kind of burden‑shifting is one of the main contributors to regrettable substitution, highlighting the importance of generating and communicating reliable data within regulatory frameworks.

REACH requires data to be generated based on the annual market volume, with more extensive information applying to chemicals produced at > 1000 tonnes/year (t/y). Tonnage is used as a proxy for exposure [18], meaning that substances placed on the market at 1–10 t/y may lack chronic toxicity, developmental toxicity, or chronic aquatic toxicity data. If such data gaps exist for substances considered as alternatives to well-studied SVHCs or POPs, these gaps can directly undermine informed substitution decisions and delay early regulatory intervention [6, 55, 66]. As the example of BPA shows, regulatory pressure can rapidly shift market dynamics, while BPA has been extensively studied following decades of regulatory scrutiny, the volumes of BPA analogues (see Fig. 1) such as BPF and BPS increased substantially, despite far more limited data on their hazards [103]. This creates an environment where the introduction of alternatives proceeds faster than the accumulation of evidence needed to ensure their safety.

Given this, REACH should adopt enhanced data requirements for alternatives to SVHCs or restricted chemicals, regardless of the tonnage at which these alternatives are produced. Such requirements could include early screening for persistence, bioaccumulation, reproductive and developmental toxicity, and chronic aquatic effects. This could be supported by the development and use of high-throughput screening approaches, such as in silico models, which can enable hazard data to be generated early in the innovation process, at a stage when substitution decisions are still feasible and cost-effective [7]. In several sectors including agrochemicals, industry already employs computational models, predictive toxicology, and early‑phase screening tools, such as in silico genotoxicity and endocrine assays, alongside in vitro fish toxicity tests to filter candidate molecules before investing in full regulatory testing [63]. Initiatives such as the US Environmental Protection Agency’s ToxCast/Tox21 programmes similarly demonstrate the potential of such approaches to screen large numbers of chemicals across multiple biological endpoints [96]. These methods are particularly valuable where data on alternatives are limited, and decision making must proceed under uncertainty.

However, while the use of New Approach Methodologies (NAMs) is promising, important limitations remain. Complex endpoints such as repeated-dose toxicity, carcinogenicity, and chronic toxicity are not yet fully captured by existing non-animal methods, making it difficult to replace in vivo studies entirely while maintaining a high level of protection for human health and the environment. Strengthening regulatory confidence in NAMs will therefore require greater cross-substance validation, coordinated data generation, and the development of more unified assessment standards [36]. A tiered approach, combining predictive methods for initial screening with targeted in vivo or in vitro studies where required may offer a pragmatic way forward. Such an approach would allow regulators to balance efficiency with precaution, helping to ensure that alternatives do not enter the market with significant data gaps or unanticipated hazards [39].

Another way of preventing regrettable substitution is for regulatory agencies (ECHA/HSE) to establish a formal mechanism for back-reporting of data, whereby authorities can signal data reliability concerns, revised interpretations, or outstanding information requests directly on the disseminated REACH dossier webpage. Under REACH, submitted data in registration dossiers is made publicly available through the ECHA’s dissemination platform [37]. However, substances placed on the market at volumes below 1 t/y are not currently subject to REACH registration, meaning that data on such substances remains largely unavailable. The current incomplete feedback loop between regulatory evaluation and dissemination of REACH data can therefore create an additional barrier that can perpetuate regrettable substitution. Although REACH registration dossiers are publicly available through the ECHA’s dissemination platform and widely used and treated as authoritative by industry, regulators, and civil society, the platform does not systematically communicate on-going regulatory scrutiny. When authorities identify unreliable studies, request new data, or reinterpret results during substance evaluation, these conclusions are not reflected in the disseminated dossier. As a result, contested or downgraded studies may continue to appear unqualified, creating a transparency gap by allowing questionable data to influence substitution decisions. It is therefore important for regulatory authorities to signal data reliability concerns, revised interpretations, or outstanding information requests directly on the disseminated webpage. Such a mechanism would not require disclosure of confidential business information nor alter data ownership; rather, it would use standardised indicators such as annotations, symbols, or status tags linked to individual studies or endpoints. These indicators could highlight where regulatory review has raised concerns about reliability, situations where the conclusions differ from those presented by the registrant, or where additional data have been required to resolve uncertainty. Implementing this measure could significantly strengthen the integrity and usability of publicly accessible REACH data. It could support industry in making informed and preventive substitution decisions, enhance transparency for downstream users and regulators, and improve regulatory credibility. Crucially, this recommendation does not introduce new testing obligations but instead improves the interpretive environment in which existing data are used. By formally closing the feedback loop between evaluation and dissemination, back-reporting could play a direct role in preventing regrettable substitution.

### Create coherent and coordinated regulatory approaches

Use-based chemical regulation allows authorities to tailor risk management to specific sectors and develop specialised expertise. However, this structure can also create fragmented and siloed regulatory regimes, where the same chemical may be assessed differently depending on the competent authority and regulatory scope and protection goals, e.g., pesticides and biocides [45, 97]. Oltmanns et al., [73] mapped data requirements for chemical assessment under use-specific regulations and observed differences between regulations, these include variation in the endpoints addressed, the stage at which data for particular endpoints are required, and the testing methods applied. Divergences in risk assessment methodologies may also influence regulatory conclusions, including differences in the reliance on modelled versus measured exposure data, and the application of assessment factors. Such inconsistencies can lead to different regulatory conclusions and undermine confidence in regulatory outcomes. It is therefore important for regulatory agencies to create a coherent and coordinated regulatory approaches.

One way of doing this is to consider the use of consistent terms and requirements across regulatory frameworks. An evaluation performed by Andreassen et al., [3] found inconsistencies in the requirements for chemical identifiers across regulatory frameworks. For example, under REACH, the term SVHC refers to chemicals presenting serious hazards. However, plant protection products (PPPs) legislation uses the term Candidate for Substitution (CfS) for active substances meeting certain hazard criteria, including PBT, carcinogenic, mutagenic or reproductive toxicity Cat 1A/1B, or endocrine disruption according to Point 4 of Annex II of Regulation (EC) No 1107/2009 [42]. Although both systems aim to encourage substitution of substances with comparable hazard profiles, their differing terms and criteria can create unnecessary complexity. Aligning these definitions and establishing consistent identification criteria could therefore support clearer, more predictable regulatory outcomes and facilitate substitution across sectors.

A deeper structural inconsistency arises in how time‑limited market access is treated. PPP active substances are approved for limited periods (typically 7–15 years), followed by systematic re‑evaluation against current scientific and regulatory standards. This helps ensure that older approvals remain aligned with evolving knowledge and policy. In contrast, REACH provides registrants with continued market access once a dossier passes completeness checks, even though this does not constitute a safety approval [19, 41]. Subsequent scrutiny occurs only selectively through dossier or substance evaluation, or via risk management measures such as restriction or authorisation, often years after registration and constrained by regulatory capacity. As a result, substances may remain on the market for extended periods despite advances in scientific understanding or the identification of new hazards. Therefore, adopting periodic re-evaluation and time-limited registration within REACH could strengthen regulatory oversight. Proposals such as time‑limited registrations or periodic re‑registration, based on updated scientific standards, could help reduce reliance on reactive risk management and better align industrial chemical regulation with evolving safety expectations. The EU proposal to introduce a 10‑year validity period for chemical registrations, with ECHA empowered to revoke dossiers that are outdated or non‑compliant, represents an important step [50].

At the same time, recent discussions under the EU Omnibus proposal have explored moving away from routine periodic re‑assessments and renewals for many pesticide active substances [51]. While this approach may aim to improve regulatory efficiency and reduce administrative burden, it also raises potential concerns regarding the timely incorporation of new scientific evidence and the ability to systematically revisit earlier decisions. Reduced reliance on scheduled re‑evaluation could increase the risk that emerging hazards remain insufficiently scrutinised, particularly in contexts where continuous data updating and monitoring are not robustly ensured. This highlights the tension between protecting human health and the environment and reducing regulatory burden for the industry. Therefore, any shift toward time‑limited approvals or reduced reassessment requirements will need to be carefully balanced with mechanisms that ensure the continuous review of emerging evidence and the timely identification and management of risks.

Fragmentation across regulatory regimes is increasingly recognised as a systemic issue in the EU [3, 19, 97]. A single substance may undergo multiple hazard or risk assessments under different frameworks, industrial chemicals, pesticides, pharmaceuticals, biocides, and consumer product regulations, with inconsistent outcomes. These discrepancies can create inefficiencies, increase burdens for regulators and industry, and weaken regulatory predictability [3, 19, 97]. The One Substance One Assessment (OSOA) initiative, a central pillar of the EU CSS, seeks to address these challenges by ensuring that chemicals are assessed consistently and only once across relevant legislative frameworks [45]. OSOA aims to consolidate scientific data, streamline assessments, and strengthen cooperation among EU agencies such as ECHA and EFSA, thereby improving coherence and reducing duplication [38]. In practice, it seeks to address long-standing fragmentation in EU chemicals regulations, where substances are assessed multiple times under different legislative frameworks using divergent methodologies and datasets. Recent legislative developments including the creation of shared data infrastructure, the reattribution of scientific tasks, and strengthened inter-agency coordination are intended to operationalise this ambition through enhanced data interoperability and promoting more consistent scientific workflows [52–54]. In the near term, OSOA is expected to deliver more integrated and accessible information systems, particularly through shared databases and improved access to existing data across agencies, thereby supporting greater transparency and consistency in assessments. Over the longer term, the initiative also envisages more structural changes, including joint or coordinated assessments, harmonised data requirements across regulatory frameworks, and more aligned decision-making processes. While these elements remain under development, together they signal a gradual shift toward a more unified and coherent EU system for chemical assessment and risk management. If effectively implemented, this approach could support earlier regulatory action on emerging risks, improve efficiency, and reduce opportunities for companies to shift hazardous substances between regulatory silos. It may also lower compliance costs by providing clearer and more predictable regulatory expectations while better aligning chemical safety assessments with broader objectives under the European Green Deal [48]. However, significant issues remain. Differences in protection goals and risk assessment approaches across EU regulatory frameworks, the lack of common chemical identifiers and constraints related to data confidentiality are barriers [3, 97]. Crucially, regulatory decision making remains distributed across sector-specific regimes with distinct legal objectives, limiting the extent to which assessments can be fully harmonised. Without deeper structural alignment, there is a risk that OSOA will improve coordination without resolving underlying inconsistencies. Nonetheless, more harmonised assessments could strengthen incentives for safe(r) substitution by ensuring that hazard classifications, risk assessments, and regulatory signals are consistent across all uses and frameworks. A more unified system could enhance transparency and data usability by integrating datasets across agencies, standardising formats and promoting common identifiers. By enabling joint or coordinated assessments and reducing work duplication across regulatory frameworks, it could also support a more efficient allocation of regulatory resources. And could also offer industry with clearer and more stable signals, enabling strategic planning and investment in safe(r) alternatives rather than piecemeal compliance with fragmented regimes.

### Take early action and build a proactive regulatory regime

Effective chemical risk management requires regulatory frameworks to be proactive rather than reactive, taking early action guided by the precautionary and preventative principles. This implies avoiding paralysis by analysis, where gathering extensive weight of evidence is required to justify regulatory control. The examples of BPA and PFAS demonstrate that delayed actions can lead to widespread environmental risk, despite early signals of concern; regulatory action was incremental and slow, enabling widespread exposure and environmental pollution. In the EU, the first restriction on PFOS was in 2006, yet regulatory risk management for the broader PFAS class was not proposed until nearly two decades later [19, 35]. A similar delay occurred under the Stockholm Convention, where it took ten years to move from listing PFOS to the inclusion of PFOA. These patterns indicate the long-recognised need for hazards to be identified and acted upon before chemicals reach high production volumes and widespread use [40].

**Fig. 5:**
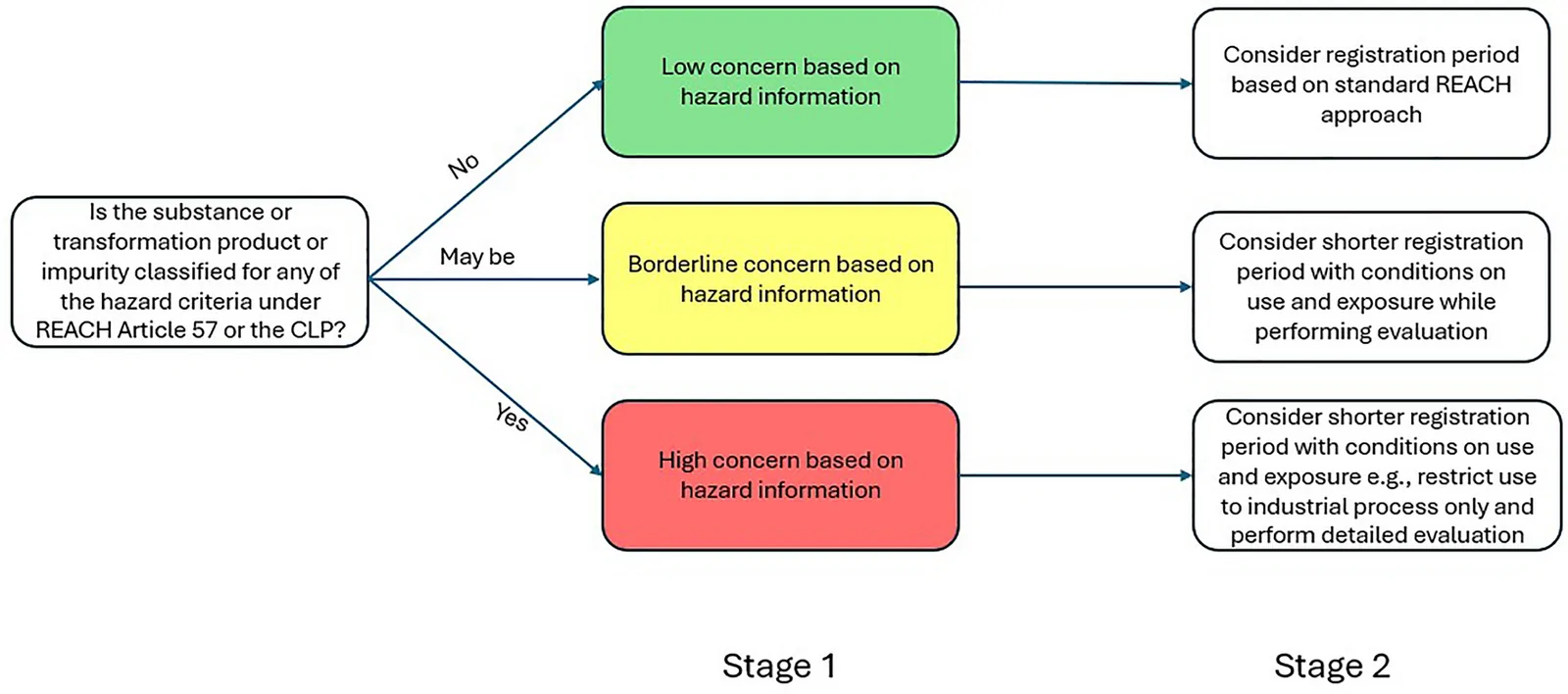
Stage gate REACH pre-registration screening. The pre-screening should be fluid and flexible to allow for substances to be easily moved between the categories depending on current understanding. Where there is data gap, consider using precautionary approach

One way of doing this is by incorporating pre‑market screening mechanisms into chemical regulatory regimes. This could involve using a stage‑gate system, see Fig. 5, in which chemicals undergo an initial hazard‑based screening before REACH registration and market access. In the first stage, a traffic‑light categorisation of green (low concern), amber (borderline concern), and red (high concern) could be applied using criteria consistent with existing REACH Article 57 SVHC definitions and CLP. This could allow regulators to identify potential priority substances early, enabling precautionary monitoring or early risk‑management consideration. In the second stage, substances in amber and red categories could be granted temporary registrations (for example, two to five years) and subjected to conditions that limit widespread exposure while further assessment is undertaken. This proactive approach could enable SVHC to be detected upstream, years before traditional substance evaluation processes would identify them, and could ensure that alternatives being considered to replace restricted or banned chemicals are also screened early to support genuinely safe(r) substitution.

The EU’s Safe and Sustainable by Design (SSbD) framework promotes the intentional and proactive (re)design of chemicals, creating an important opportunity to integrate risk management from the earliest stages of innovation [15]. As a key outcome of the EU Green Deal and the CSS, SSbD represents a pragmatic shift in how chemicals and materials are developed ensuring that safety and sustainability considerations are integrated from the start. While SSbD is presented as a much-needed transformative approach, its methodological complexity and the significant data and resource requirements, raises concern on its feasibility and usability in practice [1, 4, 98]. Although the framework was updated in December 2025 to address some of these shortcomings [58], it remains unclear whether these revisions would sufficiently resolve the underlying structural challenges. As a result, the implementation of SSbD may be uneven and risk reinforcing existing gaps in chemical risk management. Nevertheless, SSbD has a strong potential to drive the development of safe(r) and more sustainable chemicals and ultimately reduce ongoing human and environmental exposure to hazardous substances.

A proactive regulatory regime is equally required at the global level. For the Stockholm Convention to strengthen preventative chemicals management and facilitate earlier identification of substances with POP-like characteristics, Parties should be encouraged to integrate LRET screening criteria into national regulatory assessment processes particularly during substance registration, prioritisation, and alternatives assessment. Incorporating LRET at earlier stages would improve alignment between domestic regulatory systems and Stockholm Convention objectives, support more proactive risk management, and reduce the likelihood that substances with the potential for global environmental dispersion enter widespread commercial use before their long-range transport properties are recognised. Such a measure would complement, rather than replace, the Stockholm Convention’s nomination process; allowing chemicals not banned or controlled at the local level to still be nominated for global control. Parties with strong regulatory capacity could identify and act on potential POPs early, while countries with more limited capacity would retain the ability to nominate substances that are not yet controlled domestically. To illustrate, REACH which emanates from the EU and adopted by countries such as the UK, Turkey, and South Korea, is well-positioned to integrate LRET alongside existing hazard categories (e.g., (v)P(v)BT, (v)P(v)MT, CMR). Doing so could support early local and regional bans, facilitate innovation in safe(r) alternatives, and reinforce the Stockholm Convention’s objective of eliminating POP‑like chemicals from global circulation. This is especially important given that global effectiveness ultimately depends on national‑level regulatory action; when early screening is embedded in domestic systems, and hazardous chemicals are identified and controlled before they become persistent global pollutants.

### Build capacity and strengthen regulatory enforcement

Regulatory risk management of chemicals pre-REACH was widely recognised as slow and resource intensive. This culminated in the 2001 European Commission’s (EC) review which concluded that the risk assessment process is slow and resource-intensive and does not allow the system to work efficiently and effectively [40]. While REACH has since improved data availability and created more coherent procedures, the pace of regulatory action can be hampered by significant capacity and resource limitations within regulatory agencies. The REACH restriction roadmap highlights that the effectiveness of risk management, i.e., the ability to identify and control chemical risks, depends on adequate resources within the European Chemical Agency (ECHA) and Member States (MS) to perform key activities such as Risk Management Option Analyses, hazard identification, and restriction work [46]. Thus, highlighting the importance of institutional capacity, the ability of regulatory agencies to act in a timely manner remains closely linked to the resources and structures supporting implementation.

The UK faces similar challenges. Jones and Burns [65] highlight that the UK REACH system currently lacks the expertise, staff capacity, and technical capability required to keep pace with EU regulatory developments. This issue risks increasing regulatory divergence with associated implications for industry compliance, cross-border trade, and the UK’s ability to adapt to evolving scientific evidence. which may ultimately result in a slower pace of restrictions and risk management actions.

The capacity constraints are not limited to REACH. The effectiveness of the Stockholm Convention also depends heavily on Parties’ institutional and scientific capabilities to implement its obligations [2, 61, 99]. Although, the Stockholm Convention provides a robust framework for identifying and eliminating POPs, its practical impact depends on whether countries can gather the evidence required for POP nominations, participate meaningfully in the POPs Review Committee (POPRC) processes and translate global decisions into domestic implementation. Where capacity is limited, the process of identifying and restricting hazardous chemicals is often delayed, prolonging exposure and undermining the Stockholm Convention’s objectives.

Capacity limitations have direct implications not only for the speed of regulatory action but also for preventing regrettable substitution. Alternatives assessment relies on expert knowledge, quality data, and the technical ability to evaluate the comparative safety and performance of potential substitutes. The use of expert judgement means that, by its nature, it is difficult to standardise or prescribe through detailed guidance [90]. Where regulatory bodies face constraints in resources or technical expertise, the assessment of alternatives may be less comprehensive, increasing the likelihood that substitutes are not fully scrutinised and that risks of regrettable substitution persist. Strengthening scientific and technical capability is therefore essential. Developing expertise in areas such as lifecycle analysis, alternatives assessment, and the identification of emerging hazards could support a shift from primarily procedural compliance toward more proactive and precautionary risk management. Improved capacity would also help ensure that chemical data is not only collected but effectively evaluated and translated into timely and informed regulatory action, reducing the risk that available information is underutilised.

There is therefore a need to mobilise expertise beyond regulatory agencies to support timely risk management. Strengthening pathways for researchers and public-sector scientists to contribute to hazard identification and substitution efforts could help address this gap. Increased collaboration between regulatory bodies, academia, and industry would expand the available knowledge-base and support innovation in risk assessment and substitution. Increasing regulatory capacity is also critical for accelerating the pace of chemical control and for incentivising industry transition [27]. Increased capacity in terms of staffing and knowledge may help regulators to make more timely and informed decisions on hazardous chemicals and their alternatives and may also strengthen regulatory signals that can, alongside other economic and technological drivers, support earlier industry consideration of safe(r) alternatives rather than reliance on regulatory intervention alone.

Therefore, underlining the need for regulatory frameworks such as REACH and the Stockholm Convention to be supported with sustained investment in regulatory capacity, staffing, expertise and tools required if they are to effectively prevent regrettable substitution. Ensuring that regulatory ambition is matched by institutional capability is essential to achieving timely, science‑based decision‑making and ensuring that chemical substitutions result in genuine, long‑term reductions in risk. One practical way to achieve this is for policymakers and regulators to systematically involve academic experts in processes such as POPRC. This would require reducing institutional reticence toward academic input and creating clearer, more transparent mechanisms for scientific engagement. Structured participation of academia through expert consultations, commissioned reviews and collaborative research platforms can strengthen the evidence needed for risk evaluation and support the identification of safe(r) alternatives.

More so, effective chemical regulation depends not only on strong legal frameworks but also on the robustness of monitoring and enforcement systems. Without consistent oversight, even well‑designed policies risk becoming aspirational rather than operational. Monitoring and enforcement would allow regulators to determine whether restrictions, authorisation conditions, and broader risk‑management measures are functioning as intended, i.e., reducing risks to human health and the environment, improving substitution outcomes [5, 57], and ensuring a level playing field for all stakeholders. They also generate vital information on emerging risks, helping authorities adapt regulatory responses as new scientific and technical evidence becomes available.

A significant weakness within the current REACH system is that substances manufactured or imported at < 1 t/y fall outside registration requirements. This gap which leaves policymakers unaware of where and how those substances are used in commerce needs addressing by introducing a basic reporting obligation for substances used at < 1 t/y. Requiring manufacturers and importers to provide core information on function, sector of use, and product types. Such minimal reporting could provide important baseline data, enable more effective risk management and support early identification of emerging concerns.

Risk assessments often rely heavily on assumptions and estimated release concentrations, particularly when real‑world monitoring data are unavailable. Where reliable monitoring data exist, they can provide the ground‑truthing or evidence based needed to improve these approaches. At the same time, monitoring presents practical challenges. These include the difficulty in attributing observed environmental effects to specific chemicals, detecting and analysing the often-low concentrations at which chemicals occur in the environment and interpreting results in ways that can inform regulatory action. Given these difficulties, monitoring efforts should be targeted, risk-based, prioritising high‑volume and widely dispersed substances, particularly those with known risks, while maintaining the flexibility to identify emerging contaminants.

At the international level, the Stockholm Convention provides a model for global monitoring through its Global Monitoring Plan (GMP). Established under Article 16, the GMP generates harmonised environmental and human biomonitoring data on POPs, enabling Parties to track trends and assess whether control measures are achieving their intended outcomes [57, 67, 88]. Through regional monitoring networks, capacity‑building activities, and periodic synthesis reports, the GMP helps link scientific evidence to global policy decisions. However, its effectiveness remains uneven. Differences in technical capacity across regions, persistent data gaps, and challenges in monitoring newly listed or emerging POP‑like substances limit its consistency and coverage [2, 67]. While the GMP is well suited to tracking long‑term environmental trends, it is less effective in enabling rapid policy responses or anticipatory regulation. Strengthening monitoring infrastructure, improving how monitoring data are integrated into regulatory decision-making, and adopting more adaptive approaches capable of capturing emerging chemical groups could enhance both the responsiveness and the preventative capacity of the Stockholm Convention.

Effective monitoring and enforcement are equally important for driving meaningful substitution. When oversight is weak, substitution tends to remain largely voluntary, resulting in slow and uneven uptake of safe(r) alternatives [29]. Strengthening enforcement therefore requires a more integrated approach, where related regulatory frameworks such as Water Framework Directive and the Industrial Emissions Directive are considered and used as a feedback loop to the effectiveness of regulatory actions. Greater alignment across these regulatory frameworks could enhance compliance, support fair and competitive market environment, and contribute to more successful substitution outcomes [71].

### Use incentives and support to drive change towards sustainable chemical transition

The use of incentives and structured regulatory support can play an essential role in encouraging innovation and the development of safe(r) alternatives. Under REACH, the authorisation process links continued market access to a time‑limited review period, the duration of which partly depends on the applicant’s substitution plan. In principle, this mechanism is meant to stimulate substitution by making the continuation of authorised uses contingent on demonstrable progress toward adopting safe(r) alternatives [22]. In practice, however, there is no systematic regulatory engagement and follow‑up during the authorisation period. Once authorisation is granted, interactions between regulatory agencies and authorisation holders generally do not resume until about eighteen months before the review period expires, when the holder submits a re‑application. Under REACH Article 61, authorisations remain valid until reviewed by the Commission, provided the holder submits a report at least eighteen months before expiry, including an updated analysis of alternatives and, where relevant, a substitution plan. This absence of interim engagement means that regulators remain largely unaware of actual progress achieved, challenges encountered, or any deviation from the original substitution plans.

Authorisation is an important regulatory tool, particularly given that not all hazardous chemicals can be substituted in the short or medium term. It allows continued use of hazardous substances where risks can be adequately controlled or where socioeconomic benefits outweigh the risks. Yet without structured, regular touchpoints and follow‑up, the process risks functioning more as a static end-of-line permission than as an active driver of innovation. To ensure that authorisation remains a dynamic instrument for promoting substitution, regulatory agencies should establish a formal feedback loop between regulators and authorisation holders, consisting of proportionate, regular touchpoints during the review period. These touchpoints should assess progress against substitution plans, identify emerging technical or economic barriers, and clarify whether additional guidance or regulatory support is needed. For example, the proposed EU Substitution Centre aiming to support businesses in replacing hazardous chemicals could provide an avenue to assign an authorisation holder to a given centre for progress checking and support provision. Importantly, their frequency should be tailored to the complexity of the use and the feasibility of available alternatives. This approach could provide regulators with real‑time insight into substitution progress and offer companies constructive, practical engagement rather than retrospective assessment at the point of re‑application.

Increasing the visibility of existing alternatives is another important mechanism for accelerating substitution. Regulatory agencies already gather substantial information on potential alternatives during authorisation, restriction, and related processes under the Stockholm Convention, yet this information remains difficult for industry and other stakeholders to identify and use. Therefore, regulatory agencies should increase public communication by highlighting alternatives to Annex XIV and restricted substances and ensuring this information is readily accessible. The publication of the main alternatives to Annex XIV substances by ECHA represents an important step [23], but broader dissemination and regular updating are needed. Such disclosure should exclude confidential business information but include chemical names, functional groups, uses or sectors, and technical functions. Making alternatives visible could reduce duplicated effort across the industry, improves transparency, and supports more rapid decision‑making on substitution.

Chemical regulations are a strong driver of innovation strategies within industry. In practice, regulations can create a two-tier landscape of frontrunners and slow-to-respond. Frontrunners firms proactively respond to regulatory signals, often substituting hazardous chemicals well before regulatory actions become mandatory. They internalise regulatory risk and align innovation activities with anticipated restrictions or evolving market expectations [70, 85]. Such organisations are motivated by strategic advantages which include enhanced corporate reputation, reduced long-term compliance costs, improved market access and corporate sustainability goals [29, 85]. Contrarily, slow-to-respond firms tend to defer actions until regulatory intervention becomes unavoidable such as inclusion in REACH Annex XIV, POP Annex, restriction or ban. ECHA’s survey data illustrate this, where nearly one‑fifth of surveyed companies reported substituting chemicals only after a REACH restriction entered into force, while a further fraction acted only after listing on the Candidate or Authorisation Lists. Customer pressure and internal sustainability strategies, although influential for some, were less frequent triggers [29]. This disparity can create an imbalance in which frontrunners bear the early costs and risks of innovation, while slow-to-respond firms benefit from delayed action and from knowledge generated by others. To correct this imbalance, the use of targeted policy incentives such as risk‑based environmental levies, differentiated compliance costs, or application of the polluter‑pays principle to continued use of hazardous substances should be considered. These measures could discourage strategic delay, make early substitution economically advantageous, and more fairly distribute the costs of transitioning toward safe(r) chemicals.

Another important source of delay in substitution decision‑making could arise from the limited incentives for alternative providers and other third parties to participate meaningfully in regulatory consultations. Regulators often rely on these submissions to evaluate technical feasibility and availability of alternatives, yet third‑party engagement varies considerably. To this end, there is a need to strengthen incentives for alternative providers by offering targeted support such as certification schemes, regulatory recognition, or assistance in overcoming market and adoption barriers to encourage their participation in public consultations. These measures could ensure that regulators receive timely and relevant evidence that may help reduce delays in restriction and authorisation decisions and foster a more robust innovation towards safe(r) alternatives.

Crucially, the effectiveness of incentives depends on regulatory certainty and clarity. Innovation flourishes only when companies have confidence that investments in substitution will be recognised and rewarded with stable market access and predictable regulatory outcomes [39]. Uncertainty regarding the acceptance of alternatives or the durability of regulatory approvals can discourage innovation even among proactive firms [19]. Strengthening regulatory certainty by developing clear acceptance criteria for alternatives, offering early and ongoing regulatory engagement for chemical developers and establishing streamlined or fast-track evaluation pathways for demonstrably safe(r) substitutes can encourage broader participation in innovation [39]. Such measures could create a level playing field, reward early innovators, and ensure that regulatory frameworks support and not hinder the transition to safe(r) and more sustainable chemical alternatives.

### Build partnerships to drive effective regulatory and innovation outcomes

Preventing regrettable substitution requires policymakers to build multi‑stakeholder partnerships that bring together scientific, regulatory, and industry expertise. Effective chemical management cannot depend solely on hazard‑driven bans or isolated regulatory action; rather, it requires a coordinated innovation environment that actively supports the development, assessment, and uptake of safe(r) alternatives. Strong partnerships can create conditions under which information flows more freely, duplication of efforts can be reduced, and actors across the supply chain can jointly identify opportunities and barriers to substitution [24]. While REACH provides mechanisms that encourage communication, such as authorisation and downstream user obligations, these interactions remain limited by broader systemic constraints. Early in the implementation of REACH, registrants had more frequent opportunities for direct dialogue with ECHA, including in‑person meetings to discuss chemical safety assessments and approaches. Over time, these interactions shifted toward predominantly written and online formats. While more efficient and accessible in some respects, these formats may limit opportunities for iterative, real-time exchange, clarification of complex technical issues and the development of shared understanding between regulators and registrants. Written communication, in particular, can constrain informal feedback and reduce scope for exploring alternative assessment approaches. In this context, more interactive forms of engagement including structured workshops, technical exchanges, and, where feasible, face-to-face meetings could support more collaborative problem-solving. Given practical constraints such as geographic centralisation of regulatory agencies, such interaction does not need to rely solely on physical meetings but could be fostered through hybrid formats that enable richer dialogue. Strengthening these channels would support ongoing engagement with supply-chain actors and other stakeholders, helping to clarify challenges in risk assessment and substitution and address them more effectively.

An example of collaboration is the use of Substance Information Exchange Forums (SIEFs) under REACH, which enabled registrants to share data, avoid unnecessary duplication of studies, and develop a more harmonised understanding of hazards and risks. Similarly, the proposed EU Substitution Centre has the potential to become a focal point for collaboration (RISE, n.d [78]); by enabling systematic exchange of hazard data and lifecycle assessments for alternatives, it could also provide structured channels for dialogue between regulators and innovators, helping to ensure that regulatory expectations under REACH align with the pace and direction of development. Enhanced communication of this kind can help industry actors navigate the complexity of substitution [72] and reduce both the financial and logistical burdens associated with identifying and testing alternatives.

Partnership challenges extend beyond European regulatory frameworks to global conventions. Under the Stockholm Convention, Article 9 requires Parties to promote information exchange and cooperation. However, communication remains fragmented across jurisdictions and regulatory silos, where disparities in technical capacity and access to toxicological data, particularly between developed and developing countries, limit shared learning and hinder informed decision‑making [2, 81, 93, 100]. Furthermore, experiences with substitution, including failures and unintended consequences, are not systematically captured or shared which may increase the risk of regrettable substitution [6, 8]. Although stakeholders can contribute to Stockholm Convention processes, the involvement of innovation actors and downstream users remains relatively limited and often indirect, weakening the feedback loop between regulatory objectives and chemical design and slowing the translation of hazard-based policy signals into safe(r) products [6, 8]. To address these gaps, the Stockholm Convention could strengthen knowledge-sharing by establishing more structured international platforms dedicated to exchanging information on substitution outcomes for POPs, including both successful and unsuccessful cases. In parallel, the Stockholm Convention could build on existing processes to facilitate more systematic and meaningful contributions from innovation actors, downstream users, and technical experts particularly during risk profile and risk management evaluation stages, thereby strengthening the feedback loop between regulatory objectives and chemical design [72, 94].

Communication and partnership barriers also occur within industry, where chemical innovation often remains organised in disciplinary silos, with chemists, toxicologists, exposure scientists, and sustainability experts working largely independently. This separation can limit the integration of safety considerations at the design stage, increasing the likelihood that alternatives introduced to the market are later found to be hazardous. To reduce the risk of regrettable substitution and support the development of safe(r) alternatives, policy frameworks could more explicitly promote cross-disciplinary collaboration through targeted measures. These may include the integration of SSbD principles into research and innovation funding criteria and the development of guidance and training programmes that emphasise interdisciplinary approaches. In addition, creating platforms for knowledge exchange such as industry–regulator workshops, sectoral networks, and shared data environments could strengthen both internal and supply-chain communication. Such measures would help align industrial innovation with the objectives of the Stockholm Convention and advance SDG17 (Partnerships for the Goals) by embedding collaboration within chemicals management practices. More broadly, they underline the importance of coordinated partnerships across regulatory agencies, industry and scientific communities. Strengthened collaboration, supported by institutional mechanisms, can reduce duplication, improve knowledge sharing and help ensure that the development and implementation of safe(r) alternatives are informed by an integrated and transparent evidence base.

### Strengthen regulation through improved policy measures

The authorisation and restriction elements of REACH promote the substitution of the most harmful chemicals. Yet, in practice, decision-making is often hindered by late receipt or lack of information on alternatives. When essential information on potential substitutes, uses, and exposure is provided only at the point of public consultation or even later, regulators could face significant challenges in designing effective and proportionate risk management measures e.g., transition periods, derogations, and phase‑out. The ongoing universal PFAS restriction proposal illustrates this challenge. PFAS manufacture, use, and placing on the market would be prohibited above specified thresholds, with exemptions only where technically or economically viable alternatives are lacking. Transition periods of five to twelve years were proposed, while essential uses in fields such as medicines, PPPs, and biocides are excluded [22, 34, 35]. Which highlight the need for the adoption of policy measures that could secure early access to information on alternatives, uses, and exposure, ensuring that authorisation and restriction processes are informed from the outset. Earlier access to such information could allow regulators to set transition periods more accurately, prioritise uses where rapid action is feasible, and ensure that risk management measures are appropriate.

Exposure and lifecycle information remain equally key for informed substitution and regulatory risk management. Although REACH Article 37(2) requires downstream users to provide upstream suppliers with information needed to include their uses in exposure assessments, the information supplied is often minimal and fails to reflect site‑specific conditions. Registrants must therefore depend on generic assumptions that may not correspond to real‑world practices, potentially increasing the risk of regrettable substitution when substitutes are introduced under uncertain exposure conditions [19]. The substitution of brominated flame retardants with OPFRs such as TDBPP demonstrates how incomplete exposure assessment can lead to unintended consequences [66]. Indicating the need to consider lifecycle impacts when evaluating alternatives. At present, unlike authorisation, REACH registration does not require downstream users to submit site‑specific exposure information directly to regulators [31]. These differences indicate the need for downstream users, as a minimum, to confirm whether upstream exposure assessments accurately represent their site conditions, and where they do not, to submit site‑specific exposure scenarios or be required to provide more detail information that will ensure exposure assessment is close to reality.

Effective substitution requires not only understanding what a chemical does (its function) but also how well it must perform to be viable in a specific application. While REACH requires registrants to describe the function and sector of use, these descriptions are often too generic to support timely and informed regulatory decisions. For example, although a flame retardant may function to reduce flammability, its performance depends on factors such as how long it delays ignition, reduces heat release, or maintains structural integrity under different conditions. Without information on these performance requirements, regulators cannot fully assess whether an alternative can meet functional needs or whether exemptions or transition periods are justified. This highlights the need to expand REACH registration requirements to include more detailed information on necessary performance criteria (i.e., the technical properties required for a substance to fulfil its intended function) and relevant specifications for each use. ECHA’s authorisation guidance already emphasises the importance of including technical criteria and functional requirements [31], such detailed information becomes relevant for determining the essentiality of the use during risk management and control [105]. Extending this expectation to the registration stage would provide regulators with an earlier and more complete understanding of the performance demands that shape substitution decisions. To address confidentiality concerns, such information could be submitted as part of the follow-on registrant dossier submissions. Overall, this would enable regulators to better understand why a substance is used and help avoid narrow, drop‑in substitutions that fail to account for essential performance requirements.

Alongside these improvements, chemical grouping has emerged as a powerful regulatory tool for accelerating risk management and preventing regrettable substitution. Grouping assesses substances collectively by class, structure, function, or use rather than individually, offering more efficient oversight and reducing opportunities for drop‑in substitutions involving closely related chemicals [29]. This approach has been central to several major regulatory initiatives, including group‑based management of PFAS, bisphenols, dioxins, and asbestos [35, 102]. The EU’s universal PFAS restriction proposal is a notable recent example arising from the CSS [45], reflecting the Commission’s adoption of grouping as an interim but strategic measure. The EU restriction roadmap similarly prioritises entire chemical groups such as carcinogenic, mutagenic and reprotoxic substances (CMRs), endocrine disruptors, (v)P(v)BT), immunotoxicants, neurotoxicants, and respiratory sensitisers for broad group restrictions across all uses [45, 46].

Grouping can help prevent regrettable substitution by discouraging substitution within the same chemical class, but its scope is not unlimited. Grouping provides a mechanism for preventing regrettable substitution since it can be used based on the precautionary principle, where there is limited data for group members [16]. Hence, grouping should be implemented as a flexible, living regulatory approach, allowing for refinement as new evidence emerges. Chemicals within a group that demonstrate genuinely lower hazard profiles should be permitted as the case with the substitution of alkyl sulfonates indicate [82]. Minimum hazard datasets for group members are essential for this approach to function effectively, especially since risk management proposals must justify group inclusion on a case‑by‑case basis.

At the same time, grouping approaches present challenges for industry innovation. When entire class of substances are targeted for restriction or phase-out, companies may need to implement alternatives within relatively short timeframes to maintain business continuity. The rapid substitution of PFOS/PFOA illustrates these pressures [19]. From an industry perspective, such timelines can constrain the thorough assessment of alternatives and increase the perceived risk of regrettable substitution.

However, this concern should be considered in the context of the rationale for grouping, which aims to address structural risks associated with managing substances on a case-by-case basis and to avoid incremental substitution within hazardous chemical classes. In this sense, grouping can also reduce the risk of regrettable substitution by discouraging regrettable within-group substitutions that retain similar hazard profiles. The challenge therefore lies not only in the speed of substitution, but in ensuring that regulatory frameworks provide sufficient guidance, data, and transitional support to enable the identification of safe(r) and more sustainable alternatives.

## Conclusions

The findings of this study show that preventing regrettable substitution requires more than simply identifying hazardous chemicals. It calls for regulatory systems that are forward-looking, coherent, and capable of guiding innovation toward genuinely safe(r) alternatives. Analysis of REACH and the Stockholm Convention demonstrate that regrettable substitution stems not only from scientific uncertainty, but also from structural and procedural features of regulatory design. These include fragmented regulatory frameworks, delays in hazard identification, gaps in exposure and performance data, uneven incentives for innovation, and limited communication across supply chains and international systems. Addressing these challenges requires strengthening regulatory frameworks in several key ways. These include introducing early hazard screening mechanisms, improving data availability and transparency, coordinating assessments across regulatory silos, and embedding precautionary action before chemicals become widely used in commerce.

At the same time, preventing regrettable substitution depends on effective collaboration, robust enforcement, and sustained regulatory oversight. Strong partnerships between regulators, industry, scientific experts, and civil society are essential to ensure the flow of information needed to evaluate alternatives, anticipate emerging risks, and avoid duplication of efforts. Enhanced monitoring and enforcement can generate real‑world evidence to refine risk assessments and promote consistent compliance. Meanwhile, targeted support, incentives and regulatory clarity can encourage innovation and investment in safe(r) alternatives. By embedding transparency, adaptability, and cross‑sectoral cooperation into regulatory practice, frameworks such as REACH and the Stockholm Convention can help break the cycle of replacing one harmful chemical with another. This would support a shift from a reactive approach to risk management toward one that actively steers substitution away from hazardous drop‑in replacements and toward safe(r) and more sustainable solutions. While these measures are unlikely to eliminate regrettable substitution entirely, they represent an important part of a broader regulatory strategy to reduce its occurrence and improve decision-making in chemical substitution. However, further research is essential, in particular there is a need for systematic, evidence-based evaluation of early screening mechanisms and their effectiveness in reducing regrettable substitution.

For regulators and policymakers, these findings highlight the importance of early hazard screening, creating regular touchpoint with authorisation holders, improved data flows, and adaptive risk‑management that can support risk management of chemicals. For industry, the study shows the value of transparent hazard and performance data and cross‑functional innovation, which can help anticipate regulatory expectations and avoid costly future transitions. Ultimately, preventing regrettable substitution will depend on strong political commitment from governments, regulatory authorities and industry. By ensuring that chemical substitution is approached more carefully, transparently and responsibly, it is possible to reduce future risks, support sustainable innovation and move toward a more resilient and health-protective system of chemical management.

## Data Availability

The datasets used and/or analysed during the current study are available from the corresponding author on reasonable request.
